# Bridging the Gap Between Research and Policy and Practice: Comment on "CIHR Health System Impact Fellows: Reflections on ‘Driving Change’ Within the Health System"

**DOI:** 10.15171/ijhpm.2019.46

**Published:** 2019-06-17

**Authors:** Martin McKee

**Affiliations:** Department of Public Health and Policy, London School of Hygiene and Tropical Medicine, London, UK.

**Keywords:** Impact, Evidence, Policy, Knowledge Translation, Knowledge Brokering

## Abstract

Far too often, there is a gap between research and policy and practice. Too much research is undertaken with little relevance to real life problems or its reported in ways that are obscure and impenetrable. At the same time, many policies are developed and implemented but are untouched by, or even contrary to evidence. An accompanying paper describes an innovative programme in Canada to help bridge this gap. This commentary notes the growing acceptance of such initiatives but highlights the challenges of sustaining their benefits.

In the summer of 1967 the city of Detroit erupted in violence. In the course of a single week, 43 people were killed and over 2000 buildings were destroyed. The predominant view, in statements by politicians and media coverage, was that this was simply a reflection of immaturity and deviancy among the African-American population involved in the riots. A professor of psychology at the University of Michigan, Nathan Caplan, was not so sure. He went to the neighbourhoods worst affected by the rioting, even while it was still taking place along with a local journalist, Philip Meyer. Within a week they had recruited and trained 30 African-American researchers to undertake a survey. Within a month, the results were analysed and published.^1^

Today, the importance of doing policy relevant research is widely accepted. Then, the reaction was very different. Caplan describes how: *“My academic colleagues had a habit of interpreting reality as though it’s just a special case within theory. God forbid that anything they did became useful or that they actually spoke to anybody.”*^2^ His findings flatly contradicted many of the assumptions that had been taken for granted. The likelihood that someone would riot was not, as had been assumed, associated with economic status, education or, as many believe, recent migration from the southern states. Instead, it reflected experience of police brutality, overcrowding, and lack of jobs. The findings were very influential in the work of the National Advisory Commission on Civil Disorders, or Kerner Commission, set up by President Johnson to investigate the race riots that took place that year. Later, Meyer would write what has become a seminal text on the use of social science methods for journalists.^3^

There were, however, even then researchers who bemoaned the lack of contemporary relevance of much research. Indeed, as one paper at the time noted, it almost seemed as if researchers did not want anyone to read what they were writing, producing texts that were essentially incomprehensible to anyone outside their discipline,^4^ communicated in conferences attended by a few like-minded individuals, only appearing in print years later.

The attitudes that Caplan confronted still, however, persist in some quarters. Of course, there will always be research whose importance is unclear at the time. As long ago as 1892 a correspondent in the journal Nature noted how the importance of some research may only be recognised much later, asking “if universities do not study useless subjects, who will?”^5^ Yet, even if the research is speculative, that is no excuse for failing to communicate it. Too often papers are written in language that seems designed to render the findings obscure, producing results that emerge years after the problem they were intended to address, if indeed there ever was an actual problem, has been solved. Even if those involved say that they want their research to have an impact in the real world, they are unwilling to set aside time to engage with those who might use it. And while the situation has improved in recent years, even those researchers who see the importance of engagement face substantial barriers to doing it.^6^

The inaccessibility of research findings matters. Policies and practices are often enacted either in apparent ignorance of the evidence or even in direct opposition to it. The examples are numerous, with the experience of a single country, the United Kingdom, justifying an entire book filled with examples tellingly titled “The blunders of our governments.”^7^

The problem, as Lindblom and Cohen have noted, is that “… in public policy-making, many suppliers and users of social research are dissatisfied, the former because they are not listened to, the latter because they do not hear much what they want to listen to….”^8^

The accompanying paper by Sim et al describes a novel attempt to overcome this problem.^9^ The Canadian Institutes of Health Research have established a health system impact fellowship, co-locating postdoctoral fellows in a health system organisation and an academic institution. The goal was to enable the fellows to understand the challenges facing health system organisations, develop their competencies in working in a policy environment, and strengthen the health system’s ability to use evidence. The authors of the paper describe a range of positive experiences, but it is clear that the programme brought benefits for both the individuals concerned and the organisations in which they were working. However, they conclude with a rather concerning observation: “it is unresolved how the training fellowship will impact future academic success.”

This, surely, is the challenge. In many countries, the academic career structure is such that many of those obtaining PhDs will not, ultimately, pursue a career in academia. Many will use the skills and knowledge they have acquired in other ways, whether they are scientists, such as those in the pharmaceutical industry, to those with training in the arts, working in the culture and heritage sector. In many respects, these occupations are a continuation of what they studied at university. But many others will move into jobs that are far away from what they spent years studying.

As a result, in many countries there is an appreciable group of people with the ability to understand complex research but who are not using their skills, coexisting with an even larger number of policy-makers and practitioners whose work would benefit from much of the existing research, were it not for the large gap that divides them. What are needed are people who can close this gap. Crucially, this gap should be closed from both directions. Ideally, those policy-makers and practitioners that can use the research should have it translated for them, while the researchers that generate the knowledge should be informed of the needs of users so that they can ensure that what they are doing is actually useful.

This role is, increasingly, being recognised by research funders, and not just in Canada, who encourage practical placements within research training fellowships and who expect evidence of impact of the research they are funding. In the United Kingdom, for example, part of the assessment of universities for core government funding is based on impact case studies.^10^ It is also being recognised by some policy-makers, such as the international organisations and health ministries that have come together with universities in the European Observatory on Health Systems and Policies^11^ and its counterparts in North America and the Asia Pacific region.

In parallel, there has been sustained growth in research on knowledge translation. This has several strands that, to some extent reflect the diverse disciplinary backgrounds of those involved and which are not always as well developed as they might be. One set, led mainly by clinical epidemiologists and health service researchers, address the process of communication between researchers and practitioners and policy-makers, stressing the importance of mutual dialogue and trusted relationships, ensuring that researchers understand the questions being asked and practitioners understand what questions researchers can answer. An example is the SUPPORT project, a stepwise process of getting evidence into practice.^12^ Others reflect critiques of the concept of knowledge translation,^13^ focusing on thinking from other disciplines including philosophy, sociology, and political science, which argue for more attention to be devoted to the social construction of knowledge, power relationships,^14^ and the role of tacit and contextually specific knowledge.^15^ One of these, largely led by psychologists, focuses on cognitive biases, noting how two people given the same information may interpret it completely differently.^16^ This strand also includes research on how the dominant narratives, which often shape how people interpret evidence, are framed and propagated.^17^ Another, involving a mix of political scientists, information specialists, and public health researchers, among others, is exploring how some groups actively seek to undermine the communication of accurate evidence. These include vested corporate interests, with most attention having focused on the tobacco industry,^18^ but now recognising that its tactics are employed by others, such as alcohol, food, and soft drink manufacturers.^19,20^ More recently, they have begun to turn their attention to those who exploit concerns about health evidence, for example that relating to vaccines, for other purposes. These include pursuit of political goals, seeking to undermine trust in authorities and, hence, democratic institutions, or to exploit the money making opportunities of the internet.^21^ In these ways, the science of knowledge translation has become much more complex.

The report by Sim and colleagues shows the benefits that can accrue from a scheme to bridge the gap between research and policy. The challenge now is to find a way of sustaining it, ensuring that those employing the individuals who emerge from this programme find environments, on whichever side of the divide, that truly appreciate what they bring, as well as the creation of organisations that can occupy the middle ground, interpreting and translating the messages that should flow both ways between researchers and policy-makers and practitioners.

## Ethical issues

Not applicable.

## Competing interests

Author declares that he has no competing interests.

## Author’s contribution

MM is the single author of the paper.
